# Diallel Analysis and Heritability of Grain Yield, Yield Components, and Maturity Traits in Cowpea (*Vigna unguiculata* (L.) Walp.)

**DOI:** 10.1155/2020/9390287

**Published:** 2020-08-01

**Authors:** Emmanuel Yaw Owusu, Haruna Mohammed, Kulai Amadu Manigben, Joseph Adjebeng-Danquah, Francis Kusi, Benjamin Karikari, Emmanuel Kofi Sie

**Affiliations:** ^1^CSIR-Savanna Agricultural Research Institute, P.O. Box TL 52, Tamale, Ghana; ^2^Kwame Nkrumah University of Science and Technology, Kumasi, Ghana

## Abstract

Information on combining ability and reciprocal effects (REC) facilitates efficient utilization of genetic materials in a breeding program. This study was conducted (at the CSIR-Savanna Agricultural Research Institute, Ghana) to determine general combining ability (GCA) and specific combining ability (SCA), heritability, genetic advance, GCA, and SCA effects as well as the relationship between parents per se performance and progenies for yield components and maturity traits in cowpea. The test populations were derived using a 5 × 5 complete diallel cross of parents with different yield attributes and maturity durations. The results indicated that GCA was predominant for number of days to 90% pod maturity, plant height at maturity, and hundred-seed weight. This showed that genes with additive effects conditioned these traits. *Padi-Tuya*, *Songotra*, and IT86D-610 were identified as good general combiners for grain yield, while *Sanzi-Nya* was identified as a general combiner for developing extra-early duration cowpea varieties. Crosses *Songotra* × Sanzi-Nya, SARC-1-57-2 × IT86D-610, *Songotra* × SARC-1-57-2, and *Padi-Tuya* × *Songotra* were identified as good specific combiners for days to 50% flowering, pod length, pods per plant, pod yield, grain yield, and seeds per pod. The findings from this study provide useful information on the inheritance of early maturity and yield traits in cowpea. This can be exploited to develop high yielding and early maturing cowpea varieties as climate smart strategy to mitigate climate change via breeding methods such as pedigree selection and marker assisted backcrossing (MABC). Pedigree selection method is being used to develop varieties from the hybrid with high and significant SCA for grain yield, whereas the development of extra-early duration varieties via MABC with *Sanzi-Nya* (general combiner for earliness traits) as a donor parent is ongoing.

## 1. Introduction

Cowpea (*Vigna unguiculata* (L.) Walp.) (2*n* = 2*x* = 22) is one of the most commonly cultivated grain legumes in sub-Saharan Africa, possibly because of its relatively wide adaptation to drought and ability to give appreciable yields on low-nutrient soils, where other crops would fail. It has the ability to fix up to 240 kg·N/ha, with an *N* benefit of 60–70 kg/ha to succeeding crops in rotation on infertile soils [1]; hence, an essential component of farming systems in sub-Sharan Africa (SSA). Cowpea is cultivated mainly for its grains, which are rich in protein (20–25%), carbohydrates (65%) with low fat (1.8%) on dry weight basis. The grain is also rich in lysine and tryptophan. Fresh leaves are consumed as vegetable especially in Africa with protein content ranging from 27 to 34% [2]. The haulms of cowpea are used for high quality feed, particularly during the dry season when livestock feed is scarce, making the crop an essential and integral part of sustainable crop-livestock farming systems in the sub-Saharan Africa [3]. Cowpea, being a fast-cycle crop, can be grown successfully at least three times a year and fits well in the cropping system in the savannah ecologies of Ghana. It is widely cultivated in all ecologies and is a constituent crop in most farming systems, grown either as intercrop or relay crop, particularly in the northern parts of the country [4]. Most farmers in northern Ghana also cultivate preseason, early-maturing cultivars to provide food for workers during the main season's farming activities.

These benefits notwithstanding, cowpea yields in Ghana are low [5]; for instance, average grain yield of 1.5 t/ha has been reported [6], compared with >2.6 t/ha in South Africa and other countries [7]. This could be attributable to the use of low-yielding varieties, incidence of pests, diseases, and inadequate agronomic practices [5]. Although the international and national cowpea improvement programs have developed and released several improved cowpea varieties, there is still the need to develop more varieties which are resilient to current climatic challenges to maximize gain on farmer field [8]. Efforts to increase the threshold of cowpea yields in West Africa, particularly Ghana, require the identification of superior parental genotypes and understanding of the genetics of the crop as well as traits of economic importance. Knowledge of type of gene action, the magnitude of genetic variance, and combining ability estimates is needed to develop improved cultivars [9].

In the past, improvement of autogamous crops, such as cowpea, was achieved through random selection of parents from the naturally occurring variability, without emphasis on their genetic effects. However, the success of any crop improvement program depends on the selection based on actual performance of the parents as well as their combining ability for traits of agronomic importance [10]. Combining ability provides information about inheritance pattern of gene action to breeders for development of hybrids [11, 12]. It also plays a vital role in obtaining the genetic information on a particular trait of interest via fixed and random selection of parental lines in the shortest possible time [11, 13]. Combining ability indicates the expression of a trait, whether additive or nonadditive and the appropriate breeding strategy that will efficiently improve the trait [14]. General combining ability (GCA) is mainly attributable to the additive genetic effects, whereas those associated with specific combing ability (SCA) are attributed to the nonadditive effects (dominance and various types of epistasis) [15].

Whereas several studies have been conducted on combining ability for grain yield and yield components in cowpea and other crops [16–19], few studies have been reported on combining ability and heritability of maturity traits and their relationship with grain yield in cowpea [16]. Early maturity relative to other crops is the unique attribute that makes cowpea one of the most important climate smart crops particularly in areas of short cropping seasons. As a result, studies are needed to understand the combining ability of maturity in order to select desirable parents in efforts to develop new improved cowpea varieties that combine farmer-preferred traits with early maturity. According to Fasahat et al., selection of the right kind of parents is essential for developing populations that can transgressively segregate into desirable progeny for the traits of agronomic importance particularly yield and maturity [20].

Transgressive segregation as a result of the combined action and interaction of nonallelic genes is essential; thus, improved grain yield and early duration can be obtained by hybrid combinations. Furthermore, exploitation of heterosis in hybrids may contribute to yield stability in cowpea. The estimates of GCA and heritability provide important information in selection of parental lines that could give rise to better progenies upon crossing. Information on combining ability of parents for maturity traits and yield and yield components in cowpea would be useful for improving the crop for environments with short growing season, particularly in the Guinea and Sudan Savanna ecologies of Ghana and SSA in general. Therefore, the objectives of this study were (1) to determine the combining abilities of selected cowpea genotypes for yield attributes and maturity and (2) to estimate heritability for maturity and yield components.

## 2. Materials and Methods

### 2.1. Location of the Experiment

The research was conducted at the Council for Scientific and Industrial Research-Savanna Agricultural Research Institute (CSIR-SARI), Tamale. The CSIR-SARI is in the Guinea Savannah agroecological zone of Ghana (latitude 9°, 25′, 41 N; longitude 0°, 58′, 42 W; altitude about 183 m above sea level). The area is characterized by a monomodal rainfall pattern, which normally begins in May and ends in early October, with an average annual rainfall of about 1200 mm. The cropping season commences in mid-June and ends in October, with the rest of the season being dry. The soils of the experimental site belong to *Ferric Luvisols* of the Tingoli series, with a brown color, moderately drained, and free from concretions [21].

### 2.2. Genetic Materials

Five parental cowpea genotypes were selected based on grain and biomass yields, maturity periods, grain size, and color through participatory breeding program conducted at multiplication sites for two consecutive years in the Guinea and Sudan Savannah ecologies of northern Ghana for the study. The present study examined these five cowpea genotypes, namely, IT86D-610, SARC 1-57-2, *Sanzi-Nya*, *Padi-Tuya*, and *Songotra*, and their progenies. These genetic materials had phenotypic variation for grain yield, number of days to pod maturity, seed size, and color (Table 1). Genotypes IT86D-610 and SARC 1-57-2 are highly yielding and aphid-resistant, advanced breeding lines obtained from the International Institute of Tropical Agriculture (IITA) in Ibadan, Nigeria, and CSIR-SARI, respectively. The IT86D-610 is high yielding but lacks preferred grain appearance, that is, large grain size and white coat color. *Sanzi-Nya* is an extra-early maturing landrace preferred by most farmers in Northern Ghana because of its earliness. *Padi-Tuya* and *Songotra* are improved varieties released by the CSIR-SARI in 2008 for large grain size and *striga* resistance, respectively.

### 2.3. Development of *F*_1_ Populations and Field Evaluation

A 5 × 5 full diallel cross was made to generate 20 *F*_1_ crosses. The 20 *F*_1_ crosses and the five parents were evaluated in a randomized complete-block design (RCBD), with three replications at research field of CSIR-SARI during the 2016 cropping season at one location due to limited number of *F*_1_ seeds obtained from the crosses. Each plot was made up of two rows, each measuring 2 m. Field pests were controlled using *K*-Optimal (cyhalothrin 15 g/l + acetamiprid 20; EC) at the rate of 500 ml per ha at vegetative stage, flowering, and pod formation. Weeds were manually controlled as and when necessary.

### 2.4. Data Collection

Data were collected on the following maturity traits: number of days to 50% flowering (DFF) (days were counted from the day of planting to the day 50% of the plants in each plot had at least one flower) and number of days to 90% pod maturity (DTM) (from the day of planting to the day 90% of the pods in each plot turned yellowish). The grain yield and its related traits were also measured from five randomly selected plants from each plot. Plant height (cm) at flowering (PHF) was measured from the base of each of the five selected plants to the terminal bud on the main stem, while plant height at maturity (PHM) was measured at 90% pod maturity. The number of branches at maturity (NBM) were counted from the five plants in each plot at 90% pod maturity, whereas in the case of number of pods per plant (NPPLT), pods were counted from each of the five selected plants. For number of seeds per pod (SPOD), 10 pods were randomly selected from each plot and the number of seeds was counted and averaged across the number of pods used. In the case of pod length (PODL), 10 pods were randomly selected from each plot and their lengths were measured in cm. The mean value was used to represent the plot. Hundred-seed weight (HSWT) was measured in grams from the weight of 100 randomly selected dried seeds. Pod yield (PYLD) and grain yield per plot (GYLD) were determined as average weight of pods and seeds harvested, respectively, from each plot and converted to t/ha.

### 2.5. Data Analysis

All data collected were subjected to analysis of variance using PROC GLM in SAS [22] to determine significance of genetic variability among the entries. Diallel analysis for GCA, SCA, and reciprocal effects (RE) was conducted following Griffing's Method 1 and Model 1 [23] and the DIALLEL-SAS05 SAS program developed by [24]. A fixed model (Model 1) was used because parents used were not randomly selected from a segregating population. Parents were selected on the basis of their prior performance. The following statistical models were used for combining ability analysis:

(1) $$\begin{array}{c} Y_{ijk}=\mu +g_{i}+g_{j}+s_{ij}+r_{ij}+b_{k}+e_{ijk}, \end{array}$$

(2) $$\begin{array}{c} r_{ij}=m_{i}+m_{j}+nm_{ij}, \end{array}$$

where *Y*_*ijk*_ is the observed trait value from each experimental unit; *μ* is the overall mean; *g*_*i*_ is the GCA effect of the *i*^th^ parent; *g*_*j*_ is the GCA effect of the *j*^th^ parent; *s*_*ij*_ is the SCA effect of the *ij*^th^ cross; *r*_*ij*_ is the reciprocal effect of the *ij*^th^ cross; *b*_*k*_, is the replication effect; and *e*_*ijk*_ is the random residual effect (experimental error).

The reciprocal effect *r*_*ij*_ was further partitioned into maternal effect (*m*_*i*_) of the  *i*^th^ parental line and nonmaternal effect (*nm*_*ij*_) of the cross between the *i*^th^ and *j*^th^ parental lines [25].

### 2.6. Estimate of Variance Components

Variance components attributable to general combining ability (*σ*_GCA_^2^), specific combining ability (*σ*_SCA_^2^), reciprocal effects (*σ*_REC_^2^), and error variance (*σ*_*e*_^2^) were computed using mean squares for GCA, SCA, REC, and error extracted from the analysis of variance table. These were obtained by equating observed means squares to their expected means squares values and solving the resulting equations for the variance components [26].

The genetic variances were determined from the following equations using variance components for general combining ability (*σ*_GCA_^2^), specific combining ability (*σ*_SCA_^2^), reciprocal effects ( *σ*_REC_^2^), and error variance (*σ*_Error_^2^) according to the procedures outlined by [25–27].

Both broad-sense (*H*) and narrow-sense heritability (*h*) were calculated from the following formulae [27]:

(3) $$\begin{array}{c} \text{broad}-\text{sense heritability}\left(H\right)=\frac{\left(\sigma_{A}^{2}+\sigma_{D}^{2}\right)}{\sigma_{P}^{2}},\\ \text{narrow}-\text{sense heritability}\left(h\right)=\frac{\sigma_{A}^{2}}{\sigma_{P}^{2}}. \end{array}$$

The relative importance of GCA and SCA effects for each trait was determined following the procedure described by [28]:

(4) $$\begin{array}{c} \text{Baker's ratio}=\frac{2\sigma_{\text{GCA}}^{2}}{\left(2\sigma_{\text{GCA}}^{2}+\sigma_{\text{SCA}}^{2}\right)}. \end{array}$$

Phenotypic coefficient of variation (PCV) and genotypic coefficient of variation (GCV) were estimated according to [29] as follows:

(5) $$\begin{array}{c} \text{PCV}\left(\%\right)=\frac{\sqrt{\sigma_{p}^{2}}}{\mu }\times 100, \end{array}$$

(6) $$\begin{array}{c} \text{GCV}\left(\%\right)=\frac{\sqrt{\sigma_{G}^{2}}}{\mu }\times 100, \end{array}$$

where  *μ* = mean value of the particular trait; *σ*_*p*_^2^ = phenotypic variance; and *σ*_*G*_^2^ = genotypic variance.

Genetic advance (GA) was computed per the formula proposed by [15] as follows: GA=*ihσp*, where *i* = 1.76 (10% selection intensity), *h* = narrow − sense heritability, and *σp* = square root of phenotypic variance. Genetic advance was expressed as percentage of the mean.

## 3. Results

### 3.1. Analysis of Variance

The results of analysis of variance showed highly significant (*p* < 0.0001) difference for all the measured traits (Table 2). The mean squares due to GCA and SCA were highly significant for all the traits. The mean squares for the reciprocals also showed significant differences for all the traits except pod yield (PLYD). Maternal effects were significant for grain yield, pod yield, days to 50% flowering (DFF), and days to first flower appearance (DFFA).

### 3.2. General Combining Ability Effects (GCA)

Significant and positive GCA effects for pod and grain yields were observed for IT86D-610, while *Sanzi-Nya* had significant but negative GCA effect (Table 3). Regarding number of days to 50% flowering and number of days to 90% pod maturity, *Padi-Tuya*, *Songotra*, and SARC 1-57-2 had significant and positive GCA effects, while IT86D-610 and *Sanzi-Nya* had significantly negative GCA effect. *Padi-Tuya* and IT86D-610 exhibited significant and positive GCA effect for plant height at maturity. On the other hand, SARC 1-57-2 and *Sanzi-Nya* had negative significant GCA effect. Seeds per pod exhibited nonsignificant GCA effect for all the parents except IT86D-610. *Padi-Tuya* and *Songotra* had significant and positive GCA effect on hundred-seed weight, while parent *Sanzi-Nya* had significant and negative GCA effect. Parental genotype IT86D-610 was found to be a good general combiner for grain yield and pod maturity, while *Sanzi-Nya* was found to be a good general combiner for days to 90% pod maturity.

### 3.3. Specific Combining Ability

Positive and significant SCA effect for grain yield (GYLD) were detected from crosses of *Padi-Tuya* x *Songotra*, *Songotra* × SARC 1-57-2, *Padi-Tuya* × IT86D-610, SARC 1-57-2 × IT86D-610, *Songotra* × *Sanzi-Nya*, and IT86D-610 × *Sanzi-Nya*, while cross *Songotra* × IT86D-610 had negative significant SCA effect (Table 4). Crosses SARC 1-57-2 × *Sanzi-Nya* and IT86D-610 × *Sanzi-Nya* were the best crosses for days to 50% flowering. The crosses *Padi-Tuya* × *Songotra*, SARC 1-57-2 × IT86D-610, and *Padi-Tuya* × *Sanzi-Nya* recorded significant but negative SCA effect for days to pod maturity while the rest of the crosses recorded nonsignificant SCA for days to pod maturity. Crosses with positive and significant SCA effect for number of branches at maturity were *Padi-Tuya* × SARC 1-57-2, *Songotra* × SARC 1-57-2, *Songotra* × IT86D-610, and SARC 1-57-2 × IT86D-610, while cross SARC 57-2 × *Sanzi-Nya* had significant and negative SCA effect. Plant height at maturity showed significant and positive SCA effect from crosses *Padi-Tuya* × *Songotra*, *Padi-Tuya* × SARC 1-57-2, *Songotra* × SARC 1-57-2, and *Songotra* × *Sanzi-Nya*. In contrast, crosses *Padi-Tuya* × IT86D-610, *Songotra* × IT86D-610, P3x IT86D-610, *Padi-Tuya* × *Sanzi-Nya*, and SARC 1-57-2 × *Sanzi-Nya* showed negative and significant SCA effect for plant height at maturity. The SCA effect for pod length was not significant (*p* > 0.05) for the crosses except cross IT86D-610 × *Sanzi-Nya*, which had positive significant SCA effect. The number of seeds per pod had positive and significant SCA effect for cross *Songotra* × *Sanzi-Nya* and IT86D-610 × Sanzi-Nya, while cross *Padi-Tuya* × *Sanzi-Nya* and SARC 1-57-2 × *Sanzi-Nya* had negative and significant SCA effect for number of seed per pod. Hundred-seed weight exhibited positive and significant SCA effect for only cross *Songotra* × *Sanzi-Nya*, while only cross IT86D-610 × *Sanzi-Nya* had negative and significant SCA effect.

### 3.4. Estimation of Genetic Parameters

The variance components due to SCA were predominant over GCA and REC variances for most of the measured traits (Table 5). The magnitudes of SCA variance values were greater than GCA variance for all traits except number of days to 90% pod maturity, plant height at maturity, and hundred-seed weight. Baker's ratio was close to 1 for number of days to 90% pod maturity (0.75), plant height at maturity (0.72), and hundred-seed weight (0.99).

Broad-sense heritability for all measured traits ranged from 40.06% to 97.99% for pod yield and hundred-seed weight, respectively, whereas narrow-sense heritability varied from 2.01% to 97.30% for pods per plant and hundred-seed weight, respectively (Table 5). Again, at least 83% broad-sense heritability was recorded for hundred-seed weight, days to pod maturity, plant height at maturity, days to 50% flowering, and plant height at flowering, whereas moderately high heritability (broad sense) estimates were recorded for pod per plant (64.36%), number of branches at maturity (63.71%), and grain yield (54.64%). Contrarily, less than 50% broad-sense heritability estimates for seeds per pod, pod length, and pod yield was recorded.

Traits such as grain yield, pod yield, number of branches at maturity, pod length, pods per plant, and seeds per plant had very low narrow-sense heritability estimates between 2.01 and 25.49 (Table 5). The magnitude of the difference between broad-sense and narrow-sense heritability ranged between 0.69 and 62.35% for hundred-seed weight and pods per plant, respectively.

Genetic advance as percentage of mean ranged from 1.64 to 61.62% for pods per plant and hundred-seed weight. Number of days to 50% flowering (5.49%), pod length (4.97%), pod yield (2.82%), seeds per pod (2.51%), grain yield (2.30%), and pods per plant (1.64%) exhibited low magnitude of genetic advance as a percentage of mean (<10%). On the other hand, number of branches at maturity had moderate estimates of genetic advance greater than 10%. High genetic advance as percentage of mean (>20%) was recorded for plant height at flowering, plant height at maturity and hundred-seed weight (Table 5).

## 4. Discussion

Griffing's analysis of variance revealed significant GCA and SCA effects among diallel crosses with both additive and nonadditive gene effects being important for the inheritance of the pod yield and maturity traits studied in cowpea. The significant positive GCA for yield and yield-related traits observed for IT86D-610 indicates that this parental line could be useful for contributing favorable alleles for improving grain yield. Significant GCA effect is a manifestation of additive and additive × additive gene interactions, which represents a heritable portion of genetic variation which is transmittable from the parent to its progeny [15]. This therefore could imply that early generation selection would be effective due to small environmental effects [16, 30]. The significant negative GCA observed for IT86D-610 and *Sanzi-Nya* for days to 50% flowering and days to 90% pod maturity also suggests that these parents could contribute favorable alleles for breeding early maturity in cowpea. Similarly, IT86D-610 and *Sanzi-Nya* are good general combiners and sources of genes for early and extra-early maturity in cowpea, respectively. Negative GCA for days to 50% flowering and days to 90% maturity are indication of favorable dominant or partial dominant alleles, which contribute to earliness [20, 31]. Therefore, breeding methods such as pedigree selection, marker assisted backcrossing, recurrent selection, or marker assisted selection could be used in effective introgression earliness gene in *Sanzi-Nya* into elite varieties [32]. This result agrees with the findings of earlier studies [17, 21, 33] which found that negative and significant GCA effect for maturity traits are useful indicators for earliness and further suggested that this could be exploited for breeding early maturing cowpea lines. Furthermore, significant positive GCA effects for *Padi-Tuya*, *Songotra*, and IT86D-610 imply that crosses involving these varieties had superior mean performance than the general mean and as such indicate evidence of desirable gene flow from the parents to the offsprings at high intensity [34]. They could therefore be used as parents to improve other genotypes particularly low yielding, but pest and diseases resistant cowpea varieties to maximize genetic gain.

The positive and negative significant SCA observed for grain yield and pod maturity, respectively, provide opportunity for improving yield and earliness in the cowpea genotypes evaluated. This was evident in the small SCA values observed for days to 50% flowering and days to pod maturity. Crosses *Padi-Tuya* × *Songotra*, *Songotra* × SARC 1-57-2, *Padi-Tuya* × IT86D-610, SARC 1-57-2 × IT86D-610, *Songotra* × *Sanzi-Nya*, and IT86D-610 × *Sanzi-Nya* were reported to be high combiners for yield. On the other hand, *Padi-Tuya* × *Songotra* and *Padi-Tuya* × *Sanzi* were good specific combiners for days to 50% flowering and days to 90% pod maturity. These crosses could be exploited for producing desirable transgressive segregants in breeding for earliness [16].

The higher values of SCA variance for yield components compared with the GCA variance and REC variance reveal the importance of genes with nonadditive effects, whereas GCA variance for maturity traits compared with the SCA variance and reciprocal variance is an evidence for additive gene effects [35].

Baker's ratio close to unity (≤1) for the maturity traits (DFF, DFFA, DFPM, and DTM) also confirms the importance of additive gene action in modulating the expression of these traits. On the other hand, Baker's ratio close to zero for grain and pod yields implies that SCA estimates were more important. The estimates of Baker's ratio for these traits (grain and pod yields) confirmed the importance of nonadditive gene action. Thus, predicting hybrid performance based on GCA values alone will be ineffective. The predominance of nonadditive gene effects for these traits also suggests that genetic gain could only be achieved through hybridization followed by selection at advanced generations, where the genes are fully fixed and expressed, dominance is dispersed, and undesirable linkage is broken [36]. The differences between the *F*_1_ crosses and their reciprocals were significantly expressed for grain weight, pod weight, days to 50% flowering, and days to first flower appearances. This implies that the success or progress made on these traits was dependent on the direction of the cross. The high magnitude of reciprocal effect indicates that maternal effects or cytoplasmic inheritance were influential [27] for grain weight, pod weight, and earliness. To ensure that the desirable levels of expression of these traits are maintained in future, crosses must be done with parents designated according to the direction of the cross with the desirable outcome.

High broad-sense heritability observed for number of days to 50% flowering (87.95%) and days to 90% pod maturity (89.22) indicates that additive or genetic factors influenced the expression of the traits and that the traits were less influenced by environmental factors. Similar results have been reported by [31]. The high broad-sense heritability estimates mean that the phenotypes were true reflection of the genotypes for the measured traits and that selection based on the phenotypic value could be reliable. On the contrary, grain and pod yields showed moderately low broad-sense heritability estimates of 54.64% and 40.06%, respectively. This indicates that the environment influenced the expression of the traits and that there is little scope for advancement and/or improvement of these traits than the maturity traits. These results are in consonance with findings of other authors [37–39].

Information on narrow-sense heritability is of prime importance to the breeder as a measure of efficiency in selection and as an index of transmissibility of favorable additive genes from parents to their offspring's [15]. Narrow-sense heritability was very high for hundred-seed weight (97.30%) and moderately high for days to maturity (66.86%) and plant height at maturity (63.90%), suggesting that additive gene effects were primarily responsible for the genetic variation in these traits and that there is high scope for the improvement of these traits through selection. Moreover, the high narrow-sense heritability indicates that these traits can be readily improved [40]. The variation in the magnitude between broad-sense and narrow-sense heritability of the different traits indicates their levels of environmental influence [39]. The wider difference between the broad- and narrow-sense heritability for pod number per plant suggests a higher environmental influence [18] and therefore difficulty in selection compared with the traits (hundred-seed weight, pod length, and so on) which had narrow differences between broad- and narrow-sense heritability. Moreover, high heritability in the narrow-sense relates to high genetic potential, low environmental influence, and good genetic variability for effective selection of the traits. The results suggest that early generation selection will be effective for the traits [31]. Therefore, selection based on phenotypic value would be a true reflection of the breeding value.

The high genetic advance observed for plant height at flowering, plant height at maturity, and hundred-seed weight shows that additive genes controlled the expression of the traits [19]. This indicates that different approaches should be considered in order to exploit better genetic variance and achieving higher genetic gains. High narrow-sense heritability along with high genetic advance observed for hundred-seed weight and plant height at maturity is an indication of additive gene action. High narrow-sense heritability paired with high genetic advance also means that phenotypic selection of individuals, as parent for the next generation, will be more effective and reliable [41]. This further implied that a high genetic gain from selection would be anticipated. These results corroborate the findings of other authors who observed high genetic advance with high heritability estimates for yield and other related traits in cowpea [17, 37]. Specifically, [37] reported high heritability along with low genetic advance for days to maturity.

Generally, there was higher phenotypic coefficient of variation than the genotypic coefficient of variation for both maturity traits and that of the yield components. However, the yield components recorded a higher PCV: GCV ratios than those of the maturity traits. This implies that the large ratio recorded for yield components was mainly due to environmental factors rather than the genetic factors controlling the traits. On the other hand, the maturity traits which had lower PCV: GCV ratios indicate that there was less environmental influence on maturity traits than grain yield. Hence, low PCV: GCV ratios are desirable in cultivar development. Similar finding has been reported by [42] for cowpea.

## 5. Conclusions

*Padi-Tua*, *Songotra*, and IT86D-610 were identified as general combiners for grain yield. IT86D-610 and *Sanzi-Nya* were also identified as general combiners for early and extra-early maturity traits, respectively. IT86D-610 and *Sanzi-Nya* have small grain size and undesirable seed coat colors (brown and mottled); however, they could be used to improve low yielding, medium to late maturing varieties preferred by farmers, depending on the region. High broad- and narrow-sense heritability estimates were observed for number of days to 50% flowering, days to 90% pod maturity, hundred-seed weight, days to maturity, and plant height at maturity suggesting potential for improved genetic gain for these traits. Strong positive association between parental performance and GCA effects for the yield components and maturity traits studied indicated that selection can be made based on *per se* performance of parents for these traits. Though these observations were based on the results of single experiment, they provide basic insight into the inheritance of yield and maturity in cowpea which can be further tested in multilocational trials to validate their reliability in cowpea breeding programs for yield and maturity.

## Figures and Tables

**Table 1:** Codes, genotypes, maturity periods, potential yields, and sources of parents used in the diallel study.

Code	Genotype	Maturity	Potential yield (t/ha)	Source
P1	*Padi-Tuya*	75 days	2.2	CSIR-SARI
P2	*Songotra*	65 days	2.2	CSIR-SARI
P3	SARC 1-57-2	65 days	1.8	CSIR-SARI
P4	IT86D-610	65 days	2.6	IITA
P5	*Sanzi-Nya*	50 days	0.8	Landrace

**Table 2:** Mean squares for 13 traits from a 5-parent diallel analysis under optimum condition in Ghana, 2016.

Trait^‡^	Source of variation (mean squares)^†^							
	REP (Df = 2)	Entry (Df = 24)	GCA (Df = 4)	SCA (Df = 10)	REC (Df = 10)	MAT (Df = 4)	NMAT (Df = 6)	Error (Df = 48)
GYLD	0.60	2.65^*∗∗∗*^	2.75^*∗∗∗*^	3.68^*∗∗∗*^	1.57^*∗∗*^	1.10^*∗*^	1.89^*∗∗*^	0.35
PWt	1.29	3.61^*∗∗∗*^	5.74^*∗∗*^	4.34^*∗∗∗*^	2.03	3.90^*∗*^	0.78	1.08
DFF	0.52	17.30^*∗∗∗*^	44.97^*∗∗∗*^	15.41^*∗∗∗*^	8.13^*∗∗∗*^	13.97	4.24^*∗∗∗*^	0.30
DFFA	0	20.32^*∗∗∗*^	46.59^*∗∗∗*^	20.08^*∗∗∗*^	10.05^*∗∗∗*^	19.05^*∗∗∗*^	4.05^*∗∗∗*^	0
DFPM	0.17	27.13^*∗∗∗*^	96.64^*∗∗∗*^	17.89^*∗∗∗*^	8.57^*∗∗∗*^	13.37^*∗∗∗*^	5.37^*∗∗*^	1.71
NBM	0.16	4.50^*∗∗∗*^	4.84^*∗∗∗*^	4.10^*∗∗∗*^	4.70^*∗∗∗*^	7.45	2.87	0.34
DTM	0.57	59.63^*∗∗∗*^	244.24^*∗∗∗*^	35.55^*∗∗∗*^	9.87^*∗∗*^	6.33	12.22^*∗∗*^	3.31
PHF	2.92	123.51^*∗∗∗*^	323.84^*∗∗∗*^	123.46^*∗∗∗*^	43.42^*∗∗∗*^	41.42	44.75^*∗∗∗*^	4.28
PHM	9.65	165.73^*∗∗∗*^	628.74^*∗∗∗*^	101.75^*∗∗∗*^	44.50^*∗∗∗*^	51.88	39.58^*∗∗∗*^	3.28
PODL	0.76	4.84^*∗∗∗*^	8.19^*∗∗∗*^	3.47^*∗*^	4.87^*∗∗∗*^	5.78	4.26^*∗*^	1.59
NPPLT	2.97	219.48^*∗∗∗*^	54.54	340.02^*∗∗∗*^	164.92^*∗∗∗*^	190.25	148.08^*∗∗*^	29.47
SPOD	3.21	5.11^*∗∗∗*^	3.57^*∗*^	6.37^*∗∗∗*^	4.47^*∗∗∗*^	2.03	6.09^*∗∗∗*^	1.20
HSWt	2.08	35.47^*∗∗∗*^	179.40^*∗*^	5.66^*∗∗∗*^	7.72^*∗∗∗*^	9.77	6.35^*∗∗∗*^	1.22

^*∗*^, ^*∗∗*^, ^*∗∗∗*^Significantly different from zero at the 5%, 1%, and 0.1% probability levels, respectively. ^†^REP, replication; entry, number of entry; GCA, general combining ability; SCA, specific combining ability; REC, reciprocal; MAT, maternal; NMAT, nonmaternal; Error, experimental error. ^‡^GYLD, grain yield; PWt, pod yield; days to 50% flowering; DFFA, days to first flower appearance; DFPM, days to first pod maturity; NBM, number of branches at maturity; DTM, days to maturity; PHF, plant height at flowering; PHM, plant height at maturity; PODL, pod length at maturity; NPPLT, number of pods per plant; SPOD, number of seeds per pod; HSWt, hundred-seed weight.

**Table 3:** GCA effects of parents^††^ for traits^‡^ under optimum condition.

Parents^††^	Trait^‡^												
	GYLD	PWt	DFF	DFFA	DFPM	NBM	DTM	PHF	PHM	PODL	NPPLT	SPOD	HSWt
P1	0.12	0.27	0.81^*∗∗*^	1.26^*∗∗*^	1.79^*∗∗*^	−0.55^*∗∗*^	2.51^*∗∗*^	4.48^*∗∗*^	6.61^*∗∗*^	0.56^*∗*^	−1.15	0.17	2.43^*∗∗*^
P2	0.07	0.19	0.81^*∗∗*^	0.66^*∗∗*^	0.86^*∗*^	0.15	1.51^*∗∗*^	0.01	−0.69	0.23	1.39	−0.10	1.50^*∗∗*^
P3	−0.07	−0.24	0.84^*∗∗*^	0.16^*∗∗*^	0.49	0.09	1.11^*∗*^	−0.65	−1.25^*∗*^	0.13	−0.11	−0.17	0.10
P4	0.34^*∗∗*^	0.42^*∗*^	−0.53^*∗*^	−0.04^*∗∗*^	−0.21	0.52^*∗∗*^	−0.36	0.85	1.35^*∗*^	−0.07	1.35	0.50^*∗*^	−0.07
P5	−0.47^*∗∗*^	−0.65^*∗∗*^	−1.93^*∗∗*^	−2.04^*∗∗*^	−2.94^*∗∗*^	−0.21	−4.76^*∗∗*^	−4.69^*∗∗*^	−6.02^*∗∗*^	−0.84^*∗*^	−1.48	−0.40	−3.97^*∗∗*^
S. E. *g*_*i*_	0.17	0.29	0.15	0.00	0.37	0.16	0.51	0.59	0.51	0.36	1.54	0.31	0.31
S. E. *g*_*i*_ − *g*_*j*_	0.26	0.47	0.24	0.00	0.58	0.26	0.81	0.93	0.81	0.56	2.43	0.49	0.49

^*∗*^, ^*∗∗*^Significantly different from zero at the 5% and 1% probability level, respectively. ^‡^GYLD, grain yield; PWt, pod yield; DFF, days to 50% flowering; DFFA, days to first flower appearance; DFPM, days to first pod maturity; NBM, number of branches at maturity; DTM, days to maturity; PHF, plant height at flowering; PHM, plant height at maturity; PODL, pod length at maturity; NPPLT, number of pods per plant; SPOD, number of seeds per pod; HSWt, hundred-seed weight. ^††^P1, *Padi-Tuya*; P2, *Songotra*; P3, SARC 1-57-2; P4, IT86D-610; P5, *Sanzi-Nya*; S. E. *g*_*i*_, standard error of GCA effect; S. E. *g*_*i*_ − *g*_*j*_, standard error of the difference between the effects of general combining ability.

**Table 4:** Crosses^‡‡^ and SCA effects of *F*_1_ hybrid for traits^‡^ under optimum condition.

Crosses^‡‡^	Trait^‡^												
	GYLD	PWt	DFF	DFFA	DFPM	NBM	DTM	PHF	PHM	PODL	NPPLT	SPOD	HSWt
P1 × P2	0.70^*∗*^	0.73^*∗*^	−1.01^*∗∗*^	−1.66^*∗∗*^	−1.76^*∗∗*^	−0.09	−2.41^*∗∗*^	4.42^*∗∗*^	4.32^*∗∗*^	0.37	7.18^*∗∗*^	−0.07	−0.73
P1 × P3	−0.04	−0.24	−0.37	0.84^*∗∗*^	−0.39	0.48^*∗*^	1.16	4.42^*∗∗*^	4.39^*∗∗*^	−0.53	−1.99	0.00	1.00
P1 × P4	0.43^*∗*^	0.41	0.16	0.54^*∗∗*^	−0.19	−0.29	−0.87	−0.25	−1.88^*∗*^	−0.83	0.38	0.17	0.00
P1 × P5	0.58	0.45	−2.27^*∗∗*^	−1.96^*∗∗*^	−0.63	−0.05	−1.64^*∗*^	−0.39	−2.35^*∗∗*^	−0.39	−3.79	−1.77^*∗∗*^	0.07
P2 × P3	0.42^*∗*^	0.56	−0.37	−0.06^*∗∗*^	−1.29^*∗*^	0.61^*∗∗*^	−1.17	2.05^*∗∗*^	2.35^*∗∗*^	0.81	6.31^*∗∗*^	0.26	0.10
P2 × P4	−1.31^*∗∗*^	−1.77^*∗∗*^	−0.17	0.64^*∗∗*^	−0.26	1.01^*∗∗*^	−0.71	−5.11^*∗∗*^	−4.08^*∗∗*^	−0.33	−9.65^*∗∗*^	−1.07	−0.23
P2 × P5	1.54^*∗∗*^	0.96	−0.61^*∗*^	0.63^*∗∗*^	0.47	0.25	0.86	2.92^*∗∗*^	2.62^*∗∗*^	0.11	4.01^*∗*^	0.00^*∗∗*^	2.00^*∗∗*^
P3 × P4	0.80^*∗∗*^	0.89^*∗*^	−2.04^*∗∗*^	−1.36^*∗∗*^	−2.39^*∗∗*^	0.75^*∗∗*^	−3.64^*∗∗*^	−2.11^*∗*^	−2.18^*∗∗*^	0.27	8.35^*∗∗*^	−0.17	0.17
P3 × P5	−0.26	0.09	1.03^*∗∗*^	3.14^*∗∗*^	0.51	−0.52^*∗*^	0.09	−4.41^*∗∗*^	−4.48^*∗∗*^	−0.79	−5.82^*∗∗*^	−1.10^*∗∗*^	−0.60
P4 × P5	1.37^*∗*^	1.05	1.89^*∗∗*^	1.34^*∗∗*^	0.54	0.38	0.06	−2.25^*∗∗*^	−0.25	1.24^*∗∗*^	11.05	1.07^*∗∗*^	−1.27^*∗∗*^
S. E. *S*_*ij*_	0.22	0.39	0.20	0.00	0.49	0.22	0.68	0.77	0.68	0.47	2.03	0.41	0.41
S. E. *S*_*ij*_ − *S*_*ik*_	0.53	0.93	0.49	0.00	1.17	0.52	1.63	1.85	1.62	1.13	4.86	0.98	0.99

^*∗*^, ^*∗∗*^Significant at the 5% and 1% probability level, respectively. ^‡^GYLD, grain yield; PWt, pod yield; DFF, days to 50% flowering; DFFA, days to first flower appearance; DFPM, days to first pod maturity; NBM, number of branches at maturity; DTM, days to maturity; PHF, plant height at flowering; PHM, plant height at maturity; PODL, pod length at maturity; NPPLT, number of pods per plant; SPOD, number of seeds per pod; HSWt, hundred-seed weight. ^‡‡^P1 × P2, a cross between *Padi-Tuya* and *Songotra*; P1 × P3, a cross between *Padi-Tuya* and SARC 1-57-2; P1 × P4, a cross between *Padi-Tuya* and IT86D-610; P1 × P5, a cross between *Padi-Tuya* and *Sanzi-Nya*; P2 × P3, a cross between *Songotra* and SARC 1-57-2; P2 × P4, a cross between *Songotra* and IT86D-610; P2 × P5, a cross between *Songotra* and *Sanzi-Nya*, P3 × P4, a cross between SARC 1-57-2 and IT86D-610; P3 × P5, a cross between SARC-1-57-2 and *Sanzi-Nya*; P4 × P5, a cross between IT86D-610 and *Sanzi-Nya Songotra* and SARC 1-57-2; S. E. *S*_*ij*_, standard error of SCA effect; S. E. *S*_*ij*_ − *S*_*ik*_, standard error of the difference between the effects of specific combining ability for two crosses.

**Table 5:** Estimates of genetic parameters^‡‡‡^ for traits^‡^ under optimum condition.

Genetic parameters^‡‡‡^	Traits^‡^												
	GYLD	PWt	DFF	DFFA	DFPM	NBM	DTM	PHF	PHM	PODL	NPPLT	SPOD	HSWt
*σ*_GCA_^2^	0.01	0.03	1.49	1.55	3.16	0.15	8.03	10.65	20.85	0.22	0.84	0.08	17.89
*σ*_SCA_^2^	0.15	0.21	2.52	3.35	2.70	0.64	5.37	19.86	16.41	0.31	51.76	0.86	0.25
*σ*_REC_^2^	0.02	0.02	0.65	0.68	0.61	0.42	1.49	6.74	6.05	0.44	19.76	0.81	0.33
*σ*_Error_^2^	0.35	1.08	0.30	0.00	1.71	0.34	3.31	4.28	3.28	1.59	29.47	1.20	1.22
*σ*_*P*_^2^	0.30	0.64	6.25	7.13	10.21	1.47	24.02	49.34	65.25	1.73	83.01	2.24	36.77
*H* ^2^ (%)	54.64	40.06	87.95	90.53	88.43	63.71	89.22	83.44	89.05	43.57	64.36	45.64	97.99
*h* ^2^ (%)	6.57	8.10	47.65	43.57	62.00	20.39	66.86	43.18	63.90	25.49	2.01	7.06	97.30
GA (%)	2.30	2.82	5.49	5.62	6.67	11.66	9.95	23.36	34.37	4.97	1.64	2.51	61.62
Baker's ratio	0.12	0.20	0.54	0.48	0.70	0.32	0.75	0.52	0.72	0.58	0.03	0.15	0.99
GCV (%)	15.74	12.96	6.08	7.01	5.693	27.83	8	27.994	28.67	7.323	37.016	13.77	35.73
PCV (%)	21.3	20.48	6.483	7.367	6.054	34.87	8.47	30.647	30.38	11.09	46.14	20.39	36.09

^‡^GYLD, grain yield; PWt, pod yield; DFF, days to 50% flowering; DFFA, days to first flower appearance; DFPM, days to first pod maturity; NBM, number of branches at maturity; DTM, days to maturity; PHF, plant height at flowering; PHM, plant height at maturity; PODL, pod length at maturity; NPPLT, number of pods per plant; SPOD, number of seeds per pod; HSWt, hundred-seed weight. ^‡‡‡^*σ*_GCA_^2^, variance of general combining ability; *σ*_SCA_^2^, variance of specific combining ability; *σ*_REC_^2^, variance of reciprocal effect; *σ*_Error_^2^ variance of random error;  *σ*_*p*_^2^, phenotypic variance; *H*^2^ (%), broad-sense heritability; *h*^2^ (%), narrow-sense heritability; GA (%), percentage genetic advance; GCV (%), percentage of genotypic coefficient of variation; PCV (%), phenotypic coefficient of variation.

## Data Availability

The data used to support the findings of this study are available from the corresponding author upon request.
